# LONG-TERM CARE IN-HOME: LIVED EXPERIENCES OF INDIVIDUALS IN VA MEDICAL FOSTER HOMES

**DOI:** 10.1093/geroni/igae098.4364

**Published:** 2024-12-31

**Authors:** Chelsea Manheim, Rachel Fix, Leah Haverhals

**Affiliations:** Rocky Mountain Regional VA Medical Center, Aurora, Colorado, United States; Rocky Mountain Regional VA Medical Center, Aurora, Colorado, United States; Rocky Mountain Regional VA Medical Center, Aurora, Colorado, United States

## Abstract

The Department of Veterans Affairs (VA) Medical Foster Home (MFH) program provides long-term care for Veterans who are nursing home eligible but desire to reside in home-like environments. In MFHs, Veterans are matched with non-VA caregivers to live in the caregiver’s home and become part of their family. As the Veteran population rapidly ages, the VA is proactively expanding the MFH program to all VA Medical Centers (VAMCs) by 2026. This work describes lived experiences of Veterans in MFHs, their caregivers, and their family members. From February-April 2024, we conducted N=23 phone interviews with these individuals from 10 sites across eight states. Sites are part of a current VA Office of Rural Health (ORH) MFH expansion program. Veterans described living fulfilling lives in MFHs and taking advantage of activities in home and in the community through assistance from their caregivers. Veterans who lived previously in nursing homes shared that their lives improved greatly once they moved into MFHs. Many caregivers found their work highly rewarding, but also shared that caregiving can be isolating and that the VA provides them with limited respite options. Therefore, those who serve as caregivers must have a great desire to do this work. Some family members described that placing their Veteran family member in a MFH allowed them to return to work and expressed deep appreciation of the program. VAMCs starting and expanding MFH programs can apply findings from this work to better design and implement MFH programs in coming years.

